# Occupational stress and its association with obesity among Healthcare Workers in Madinah

**DOI:** 10.12669/pjms.41.9.12497

**Published:** 2025-09

**Authors:** Maryam Abdullah Almubarak, Farah Asad Mansuri, Hind Abdullah Almubarak, Alanoud Nwishi Almohammdi

**Affiliations:** 1 Maryam Abdullah Almubarak, Department of Preventive Medicine, Resident Physician, Faculty of Medicine and Health Sciences, Saudi Commission for Health Specialties, 42518, Madinah, Saudi Arabia; 2 Farah Asad Mansuri, Department of Family and Community Medicine, Faculty of Medicine and Health Sciences, Taibah University, 42518, Madinah, Saudi Arabia; 3 Hind Abdullah Almubarak, Faculty of Dentistry, Taibah University, 42518, Madinah, Saudi Arabia; 4 Alanoud Nwishi Almohammd, Faculty of Dentistry, Taibah University, 42518, Madinah, Saudi Arabia

**Keywords:** Cross-sectional, Healthcare worker, Occupational stress, Obesity

## Abstract

**Objective:**

Occupational stress among healthcare workers (HCWs) is a worldwide concern despite its substantial impact on workers. It could impact the health status of the HCWs by reducing their quality of life and affecting their job performance. Additionally, HCWs are susceptible to obesity because of their work schedules and sedentary lifestyle. This study explored the relationship between occupational stress and obesity among HCWs in Al-Madinah, Saudi Arabia.

**Methodology:**

An analytical cross-sectional study was designed in primary healthcare centers in Al-Madinah, Saudi Arabia, from November 2024 until January 2025. Data was collected through a questionnaire that was randomly distributed. The questionnaire contained the Occupational Stress Index (OSI) tool to assess the stress level among the HCWs.

**Results:**

A total of 232 HCWs were enrolled in the study. More than half were females, 122 (52.8%), and 40.5% were between 30 and 40 years old. About 53.4% of the included HCWs expressed a moderate occupational stress level, and 37.7% were obese. The median OSI score was significantly higher among females than males [83 vs 80; p value 0.032]. HCWs aged 30 to 50 years and years of practice had a significant association with BMI values, with a p value of 0.001 and 0.006. There was a non-significant, nearly negligible correlation between obesity and occupational stress.

**Conclusion:**

This study found that female health workers were significantly more stressed than males. Healthcare organizations might include mandatory regular screening for obesity and mental health for their HCWs and early intervention programs.

## INTRODUCTION

Occupational stress among healthcare workers (HCWs) is a worldwide concern that has brought very little attention despite its substantial impact on workers. In Spain, it’s reported that half of the primary HCWs had occupational stress.^1^ In Saudi Arabia, a study found that 15.8% of HCWs suffered from high work-related stress.^2^ This is attributed to several reasons, including the highly demanding nature of the healthcare job and a lack of social support. Also, shift-based work, intensive workload, long working hours, and a worker shortage.^3^

Consequently, occupational stress could impact the health status of the HCW by reducing their quality of life, affecting job performance, and leading to lower satisfaction rates. It can even result in poor health outcomes such as hypertension, stroke, and depression. Moreover, it is well documented that occupational stress is associated with low energy levels, tiredness, and changes in eating habits by increasing the tendency to crave unhealthy, fat-rich food, which contributes to overweight and obesity.^4^

Obesity is another public health concern, defined as excessive fat deposition and related to significant complications including diabetes, atherosclerosis, and hypertension.^5^ Obesity is one of the leading causes of morbidity and mortality worldwide. Specifically, Saudi Arabia is suffering from an increasing trend of their obese population.^6^ HCWs are susceptible to obesity even with their medical knowledge because of their hectic, busy work schedules and sedentary lifestyle.^7^ Additionally, it has been found that HCWs who have low control and high demand in hospitals tend to be affected by changes in their body mass index.^8^

Furthermore, a systematic review revealed that the ghrelin hormone, which stimulates appetite and feeding behavior, is secreted in high levels during stressful states, considering it a stress biomarker.^9^ Particularly, the surge in cortisol hormone in stressful events stimulates the reward system in the brain, leading to higher intake of sugar and fat-rich food.^10^,^11^

The HCW’s well-being and mental health are essential for maintaining a sustainable quality of care and productivity.^12^ Although there have been studies addressing the relationship between occupational stress and obesity, there remains a significant need for research specifically targeting Saudi Arabia’s primary healthcare workforce. Our study aimed to constructively address this gap by measuring stress levels, utilizing the Occupational Stress Index, and assessing the prevalence of obesity among healthcare workers in Al-Madinah. This will contribute valuable insights to the local epidemiological data and inform potential interventions for improving the well-being of healthcare professionals. Therefore, this study explored the relationship between occupational stress and obesity among primary healthcare workers in Al-Madinah, Saudi Arabia. Also, the level of occupational stress and prevalence of obesity in the same population were assessed.

## METHODOLOGY

A cross-sectional observational study was designed to fulfil the previously mentioned objectives. The study was conducted in primary healthcare centers in Al-Madinah, Saudi Arabia, from November 2024 until January 2025. The study enrolled all healthcare workers, including physicians and nurses, practicing in primary healthcare centers in Al-Madina, Saudi Arabia.

### Ethical approval:

Ethical approval for this study was granted by the Institutional Review Board (IRB) of the General Directorate of Health Affairs in Madinah, Saudi Arabia (National Registration Number with the National Committee for Bioethics [NCBE], KACST, KSA: H-03-M-S4; Approval Letter Date: November 7, 2024; IRB Log No: 2.1-114). Informed consent in writing was secured from each participant following a thorough explanation of the study’s aims, methods, possible risks, advantages, and their ability to withdraw at any point without repercussions. All gathered data were made anonymous, and confidentiality was rigorously upheld by the Declaration of Helsinki.

### Inclusion criteria:

- Agreed to participate in the study
- Healthcare workers (physicians and nurses) in the primary healthcare centers in Al-Medina city

### Exclusion criteria:

- Medical students
- Taking medication that affects stress (antidepressants and steroids)
- Pregnant

### Sampling technique and sample size calculations:

A multistage cluster sampling technique was followed. Sixty-five primary healthcare centers in Al-Medina city were divided into four regions, and two primary healthcare centers were randomly selected. The participants were then chosen using a convenience sampling method.

Two hundred thirty-two participants were calculated using the openepi.com online calculator. Based on a 95% confidence level, a 5% margin of error, a 21% population proportion, and 2518 population size according to the statistical yearbook for Al-Medina, Saudi Arabia, 2022.^13^

### Data collection method and tool:

A questionnaire was distributed to randomly selected participants through direct communication with primary healthcare centers. The data collection included a questionnaire with two sections. The first section was demographic characteristics (age, gender, smoking, taking steroidal or anti-depressant medications, years of practice, title position, and body mass index). Obesity was assessed using the body mass index (BMI), which involved gathering the weight (kg) and height (m) measurements of the participants. The BMI was subsequently computed using the formula: weight (kg) divided by height (m)². The resulting BMI is classified: underweight (<18.5 kg/m²), normal (18.5-24.9 kg/m²), overweight (25–29.9 kg/m²), and obese (≥ 30 kg/m²). The second section was the Occupational Stress Index (OSI), which included 26 items distributed in seven subscales (role overload, unreasonable group and political pressure, responsibility for personal, powerlessness, peer group relations, strenuous working conditions, and unprofitability).

The 26 items of the OSI questionnaire included 20 true-keyed and six false-keyed (items from 23 to 28). Responses for the items keyed as true were scored as five for strongly agree, four for agree, three for neutral, two for disagree, and one for strongly disagree, whereas the items keyed as false received reversed ratings. All item scores were summed to estimate the level of occupational stress. The score was categorized as low (<60), moderate (60 to 84), and high (>84).

### Statistical analysis:

The SPSS (Statistical Package for Social Science) software version 26 was used for the statistical analysis. Descriptive analysis was performed to describe the categorical data using numbers and percentages. Numerical data were described using median and interquartile range (IQR) for not normally distributed data. At the same time, mean and standard deviation (SD) were used for normally distributed data after conducting a normality test using the Shapiro-Wilk test. A bivariate analysis assessed the associations between occupational stress, obesity, and demographic characteristics through Kruskal-Wallis and Mann-Whitney tests, in addition to Spearman’s correlation test, which was used to detect the correlation between OSI scores, BMI, and years of practice of the participants. A P-value cutoff point of 0.05 was used to determine the statistical significance. A valid percentage was obtained for missing cases.

## RESULTS

**Fig.1:**
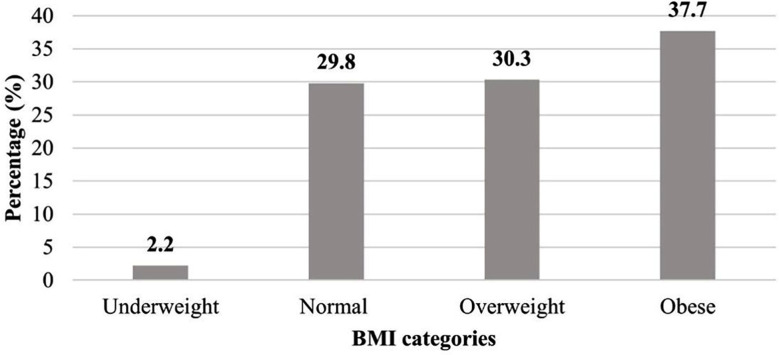
Prevalence of obesity among 228 healthcare workers.

A total of two hundred thirty-two healthcare workers were enrolled in the study after excluding one who disagreed to participate and six pregnant females. All participants confirmed that they do not take any antidepressant or steroid medications. More than half were females, 122 (52.8%), and 40.5% were between 30 and 40 years old. Most of them were non-smokers, 172 (74.1%). The highest proportion, 66 (28.4%), were nurses. They had a median BMI of 27.6 (8.4), as 37.7% were obese, as shown in Fig.1. Table-I provides more details on participants’ demographics. Regarding the OSI questionnaire, the participants’ response showed a median total score of 82 (19). The role overload, 18 (6), and peer group relations, 16 (7), showed the highest median subscale scores. This indicates that the participants strongly agreed with the work overload and strongly disagreed with being supported by their work colleagues or even having good relations with them, Table-II.

**Table-I:** Healthcare workers’ demographic characteristics (N=232).

Characteristics	Category	Number	Percentage
Age (years)	< 30	79	34.1
	30-40	94	40.5
	41-50	40	17.2
	>50	19	8.2
Gender (N=231)	Female	122	52.8
	Male	109	47.2
Smoking status	Non-smoker	172	74.1
	Smoker	60	25.9
Qualifications	Nurse	66	28.4
	General practitioner	36	15.5
	Family medicine	28	12.1
	Pharmacist	21	9.1
	Dentist	17	7.3
	Medical laboratory specialist	13	5.6
	Physical therapy	11	4.7
	Dental assistant	11	4.7
	Other*	29	12.5
*Characteristics*		*Median (IQR)*	
Years of practice		6 (12)	
Weight (kg)		75 (30)	
Height (cm) (N=228)		165 (15)	
BMI (kg/m^2^) (N=228)		27.6 (8.4)	
*Occupational stress index scores*		*Median (IQR)*	
Total score		82 (19)	
Subscale scores			
Role overload		18 (6)	
Unreasonable group and political pressure		11 (7)	
Responsibility for personal		9 (4)	
Powerlessness		9 (4)	
Peer group relations (N=225)		16 (7)	
Strenuous working conditions		13 (6)	
Unprofitability		6 (3)	

* Others: included clinical nutrition, epidemiology, internal medicine, preventive medicine, radiology, and respiratory therapy. BMI: body mass index, IQR: interquartile range.

**Table-II:** Correlation between total OSI scores and BMI.

Item	Median (IQR)	P value	Spearman’s Correlation Coefficient
Total OSI score	BMI (kg/m2)	0.514	0.043

**Fig.2:**
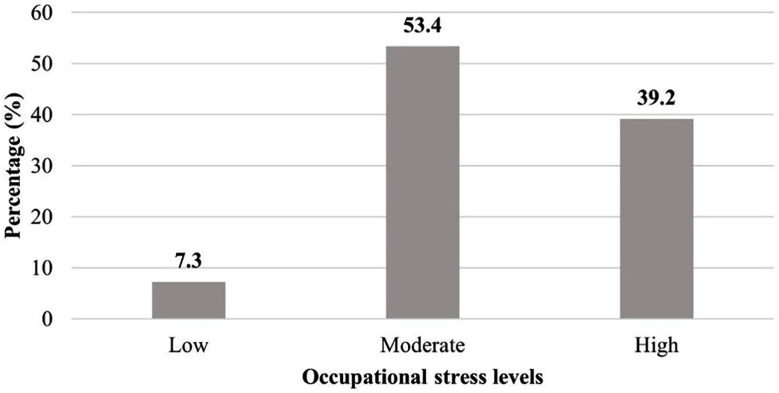
Levels of occupational stress among primary healthcare workers (N=232)

As presented in Fig.2, 53.4% of the included healthcare workers expressed a moderate occupational stress level. Table-II demonstrates the correlation between the participants’ median total OSI score and BMI. This indicates that there was a non-significant, nearly negligible correlation between them. Additionally, the association between occupational stress and demographic characteristics (Table-III) exhibited that the occupational stress was significantly (P=0.032) affecting females compared to males (83 vs 80.05, respectively).

**Table-III:** Association between occupational stress and demographic characteristics.

Factor	Category	Median OSI score (IQR)	P value
Age (years)	<30	83 (20)	0.731
	30-50	81 (19)	
	>50	81 (21)	
Gender	Female	83 (21)	0.032
	Male	80.05 (18)	
Smoking status	Smoker	83 (18)	0.559
	Non-smoker	81 (19)	
Qualifications	Physician	81 (20)	0.708
	Nurse	83 (20)	
	Allied health professionals	82 (17)	
BMI categories	Underweight	82 (23)	0.941
	Normal	81 (20)	
	Overweight	82 (18)	
	Obese	82 (19)	
Years of practice	Median (IQR): 6 (12)	82 (19)	0.279 (R=-0.071)

Moreover, the association between obesity and demographic characteristics of the included healthcare workers (Table-IV) revealed that age significantly impacts (P<0.0001) the BMI of the HCWs. Workers between 30 and 50 years showed higher BMIs than younger ones. Also, years of practice showed a very weak, significant (P=0.006) positive correlation (R=0.181) with the BMI of the HCWs.

**Table-IV:** Association between obesity and demographic characteristics.

Factor	Category	Median BMI (IQR)	P value
Age (years)	<30	24.65 (7)	P<0.001
	30-50	29.5 (9.4)	
	>50	28.7 (5.8)	
Gender	Female	28.1 (10.1)	0.332
	Male	27.23 (8)	
Smoking status	Smoker	27.5 (8.2)	0.344
	Non-smoker	27.68 (9.8)	
Qualifications	Physician	26.9 (11.5)	0.638
	Nurse	28.1 (7.3)	
	Allied health professionals	27.7 (8.9)	
Stress levels	Low	27.1 (10.6)	0.819
	Moderate	26.8 (8.3)	
	High	28.13 (9.1)	
Years of practice	Median (IQR): 6 (12)	27.6 (8.4)	0.006 (R=0.181)

## DISCUSSION

Our study addresses the gap in understanding occupational stress and its potential association with obesity among healthcare workers (HCWs) in primary care centers in Al-Madinah, Saudi Arabia. One of its main strengths is its particular focus on this understudied community, which offers insights into the Saudi Arabian healthcare workforce peculiar to the region. It confirms trends like the incidence of obesity associated with age and years of practice, and greater stress levels among females.

This literature highlighted a high rate of occupational stress incidence (23.8-87%) among the HCWs^8^, Indicating the priority of revealing the related factors and outcomes. This study identified whether there is an association between obesity and occupational stress among the HCWs of Al-Madinah, Saudi Arabia. Our study showed that 232 included HCWs had a median total OSI score of 82 (19), exhibiting a moderate level of stress (53.4%). Similarly, Thapa et al. conducted a study on 368 nurses and physicians, with a mean OSI score of 149.56±22.01.^14^ Likewise, Karen et al. revealed a 77.3 (11.8) total mean OSI score among 112 female physicians.^15^

There is no association between occupational stress and obesity in the studied group, as indicated by a non-significant, nearly negligible correlation between occupational stress and BMI in our participants (r=0.043) with P=0.514. Even though Barbosa et al. and Abolfazli et al. used different stress measurement instruments, our result is consistent with their findings (r=0.402) with P=0.084 and (r=0.023) with P>0.05. In consistent with earlier studies, most individuals also showed moderate levels of stress (64.36% in our study, 82% in Barbosa et al.).^16^,^17^

Additionally, 37.7% of the HCWs in this study were obese, and 30.3% were overweight. In the same manner, Hassanein et al. found an overweight rate of 33.8% while a lower obesity rate of 12.9% in HCWs of Al-Ahsa, Saudi Arabia.^18^

Regarding the factors affecting the OSI total stress score, our study showed that females had higher scores than males (83 vs 80.05, P=0.032), with no detected association between age and OSI total score. This confirms Thapa et al. ’s findings.^14^

On the other hand, our results showed that age significantly impacts (P<0.0001) the BMI of the participants. HCWs aged between 30 and 50 years showed higher BMIs than those younger. This is consistent with a previous study,^7^ Which could be attributed to the decline in physical activity, metabolism rate, and increased workload with advanced age.^19^ Moreover, this study did not conclude any significant association between gender and BMI of the HCW. Aligning with Kunyahamu et al.^20^ and contrary to Guo et al.^21^ Apart from this, years of practice showed a significant (P=0.006), very weak positive correlation (R=0.181) with the BMI of the HCW in this study. Dadar et al. showed an obesity rate of 29%, which was markedly associated with years of HCW practice (P=0.016).^22^

Healthcare workers frequently experience occupational stress; 53.4% of our included HCWs reported moderate levels of stress, which were mostly caused by role overload and strained relationships with peers. According to Khurshied et al, stress and burnout are major issues among Pakistani surgeons, suggesting that stress is a problem that many healthcare professionals in the area face. The lack of a significant relationship between occupational stress and BMI (r=0.043) with P=0.514 in our study, however, raises the possibility that other variables, including age and years of practice, may be more important for obesity among healthcare workers.^23^

The role overload, 18 (6), and peer group relations, 16 (7), showed the highest median subscale scores. Indicating that the participants strongly agreed with the work overload and strongly disagreed with being supported by their work colleagues or even having good relations with them. Employees who experienced a lack of social support were more likely to experience stress. This highlights that HCWs must adopt stress-reduction techniques, such as enhancing communication with their colleagues and networks at work.^24^

The high rates of obesity (37.7%) and moderate stress (53.4%) among healthcare professionals highlight the necessity of keeping an eye on and addressing both physical and mental health to preserve employee wellbeing and the standard of treatment. Important stressors like duty overload and strained peer relationships may be lessened by interventions including stress-reduction programs, improved workplace communication, and social support networks.

Our findings highlight the need for a systematic approach to support healthcare worker well-being and maintain care quality. We recommend that primary care institutions incorporate routine mental health and BMI screenings into their performance dashboards quarterly to catch early signs of burnout and unhealthy weight gain. Additionally, we suggest tiered interventions: universal stress-management workshops for new hires; confidential peer-support groups and digital wellness apps for those at moderate risk; and expedited referrals to specialists for high-risk individuals. By integrating these strategies into Saudi Vision 2030’s digital health platforms, organizations can effectively track progress, reduce absenteeism and turnover, and enhance both workforce resilience and patient outcomes.

### Limitations:

This study harbored a few limitations that could affect the final conclusion. First, this study’s cross-sectional design limits the capacity to infer causality and poses constraints, such as the possibility of biases like selection and social desirability. Furthermore, it is difficult to account for every possible confounding factor that could affect both professional stress and obesity, such as socioeconomic status, chronic illness, or personal stressors.

Second, the study’s focus on Al-Madinah’s primary care HCWs is a strength, highlighting a critical group where operational ambiguity may increase stress. However, the limited scope and sample size reduce generalizability. Longitudinal studies are recommended to explore occupational stress in diverse healthcare settings. Expanding the scope to include diverse healthcare settings across Saudi Arabia and other regions.

## CONCLUSION

More than a third of the healthcare workers in AlMadinah city in Saudi Arabia were obese, and more than half suffered from moderate occupational stress. Although this study found no correlation between occupational stress and obesity, females were significantly more stressed than males. Also, HCWs aged 30 to 50 and years of practice had a significant association with BMI values. Healthcare organizations might include mandatory regular screening for obesity and mental health for their healthcare workers, in addition to early intervention programs.

### Authors` Contribution:

**MA:** Conceived, designed, did statistical analysis, manuscript writing and responsible for the accuracy of the study.

**HA and AN:** Did data collection, critical analysis and manuscript review.

**FA:** Did manuscript review and data interpretation.

All authors have read and approved final version of the manuscript.
